# Microorganisms present in artisanal fermented food from South America

**DOI:** 10.3389/fmicb.2022.941866

**Published:** 2022-09-08

**Authors:** Maria Eugenia Jimenez, Ciara M. O’Donovan, Miguel Fernandez de Ullivarri, Paul D. Cotter

**Affiliations:** ^1^APC Microbiome Ireland, University College Cork, Cork, Ireland; ^2^Department of Food Bioscience, Teagasc Food Research Center, Fermoy, Ireland

**Keywords:** fermented food and beverages, South American food, lactic acid bacteria, fungus, microbial diversity, artisanal food

## Abstract

Artisanal fermented products (foods and beverages) are produced in an artisanal way in many countries around the world. The main purpose of fermentation is to preserve the food, improve its safety, increase the nutritional and health-promoting value and add specific flavours. In South America, there is a great variety of fermented food produced in an artisanal way. Different raw materials are used such as potatoes, sweet potato, cassava, maize, rice, milk (cow, ewe, goat) and meat (beef, goat, lamb, llama and guanaco). Some of these fermented foods are typical of the region and are part of the culture of native communities, e.g. *tocosh*, *masa agria*, *puba flour*, *charqui*, *chicha*, *champu* and *cauim* among others (indigenous foods). However, other fermented foods produced in South America introduced by mainly European immigration, such as cheeses and dry sausages, and they are also produced in many different parts of the world. In this work, the microbial composition of the different artisanal fermented products produced in South America is reviewed, taking into consideration the associated raw materials, fermentation conditions and methodologies used for their production.

## Introduction

The historic need to preserve foods out of season has resulted in the development of a variety of conservation methods. Fermentation is one of the oldest methods used for food preservation and safety improvement, being a cheap and relatively easy to apply technology for food processing that involves different modifications of food structures due to the action of various microorganisms (bacteria and fungus). The expert panel convened by ISAPP (International Scientific Association for Probiotics and Prebiotics) defined fermented foods as “*foods made through desired microbial growth and enzymatic conversions of food components”* (Marco et al., 2021).

Fermented foods are an important component of the diet across almost all societies (Hesseltine and Wang, 1980). During fermentation, a variety of metabolites and end products (such as organic acids, alcohols, peptides, amino acids, polyphenols and antioxidants) are produced as a consequence of the activities of the microorganisms present that change, sometimes radically, the texture, colour, taste, smell, or other attributes of the original food matrix improving the nutritional quality and extending the shelf life. Fermentation is also capable of increasing food quality and safety, as it prevents the spoilage and inhibits the growth of pathogens during and after the fermentation process. In general terms, this is the result of two mechanisms that work together during fermentation: the competitive exclusion of spoilage microbes, and the creation of an inhibitive environment with each microorganism influencing the others. The microorganisms responsible for fermentation produce and release large amounts of antimicrobial compounds, such as lactic, acetic and phenyllactic acids, alcohols and antimicrobial peptides inhibiting the growth of spoilage and pathogenic microorganisms while being relatively unaffected by these molecules a phenomenon termed as amensalism (Teng et al., 2021).

Many different raw materials, such as meat (including fish), vegetables, fruits, milk and cereals have been used for the production of fermented products (Wacher Rodarte, 2014; Septembre-Malaterre et al., 2018; Marco et al., 2021).

Fermented foods have been produced over centuries based on empirical knowledge transmitted from generation to generation across different countries of South America (Melatti, 1983). Fermentation has been employed not only because it is an economical way to preserve food, but because it is part of the culture of these communities and they are conventionally prepared during festivities and religious ceremonies as well as also being used in traditional medicine (Elizaquível et al., 2015). These products are generally produced on a small scale for the local population and also at a household level for consumption by individuals or families. A variety of raw materials are used to produce these artisanal fermented foods, including maize, cassava, rice, fruit, meat and potatoes (Osorio-Cadavid et al., 2008; Miguel et al., 2012; Freire et al., 2014; Elizaquível et al., 2015; Ramos et al., 2015). Most of these foods are prepared ignoring what microorganisms are present during the process (Gotcheva et al., 2000), including the microorganisms colonizing the raw materials, utensils used and the environment. These so-called “spontaneous” fermentation processes typically involve complex interactions between a combination of bacteria and fungi (Blandino et al., 2003; de Vuyst et al., 2014).

Although interest in fermented foods and beverages is growing worldwide, many artisanal fermented foods from different regions of the world are not well known or as thoroughly studied. These merit greater attention to better understand the underlying processes and to be able to harness the potential of their indigenous microorganisms.

**Figure 1:**
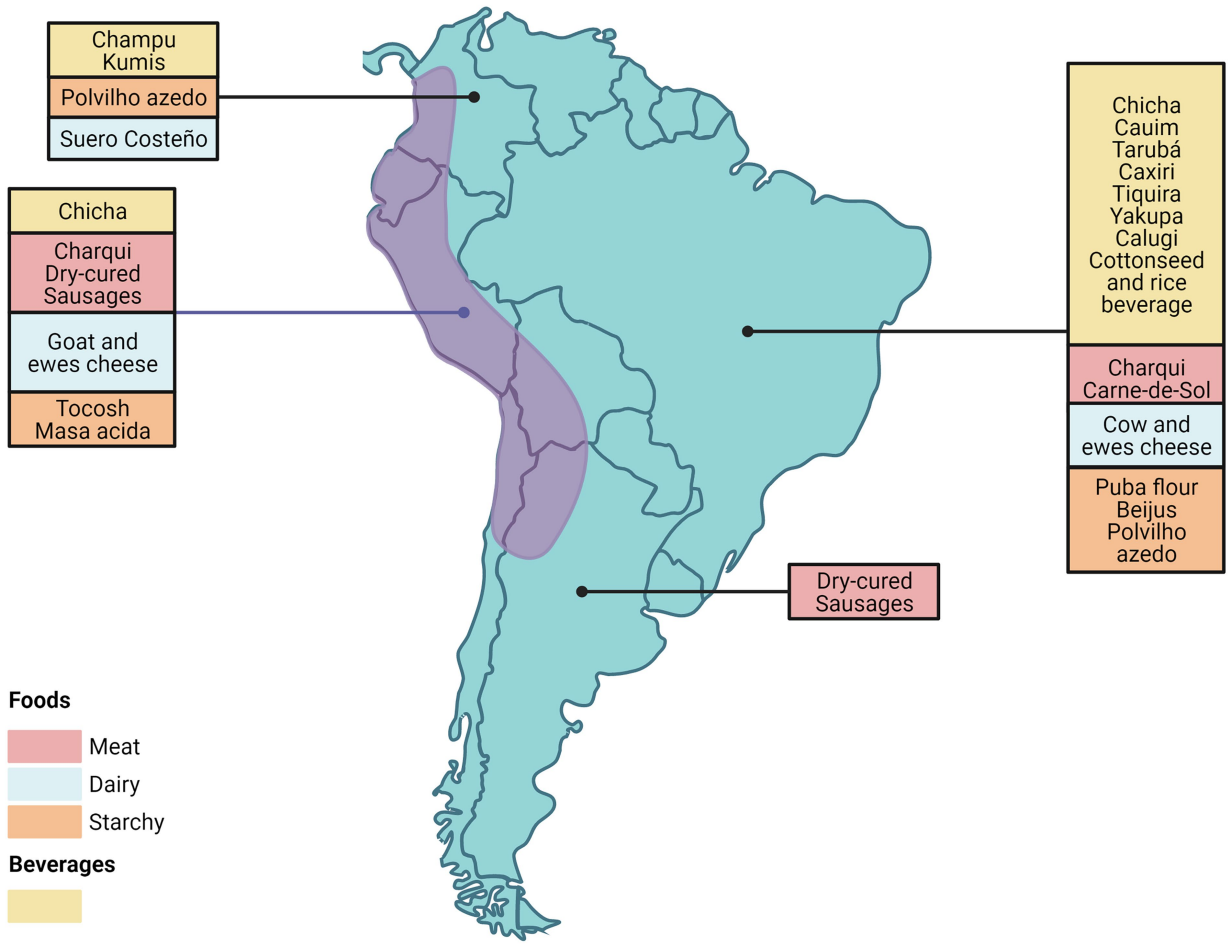
Geographic distribution of fermented food in South America. Created with BioRender.com

This review describes the diversity of microorganisms involved in the production of different artisanally produced fermented foods from South America that are typically produced on a small scale by local communities using a variety of raw materials such as cereals, fruits, vegetables, tubers, milk and meats (Figure 1). Most of the fermented foods included in this review, are South American typical indigenous foods. However, we included some foods such as cheese and salamis, which are originally from other parts of the world, and introduced by European immigration, as they are produced in a particular way or by using native ingredients in their preparation (Gänzle, 2022).

## Fermented beverages

In South America, there is a broad variety of traditional indigenous fermented beverages that continue to be made by the native populations. The beverages are prepared in a traditional and artisanal way that has been kept from generation to generation (Osorio-Cadavid et al., 2008). The microbiota responsible for the fermentation processes are most frequently a complex mixture of lactic acid bacteria (LAB) and fungi (Blandino et al., 2003; de Vuyst et al., 2014).

### Non-alcoholic beverages

#### Cauim

*Cauim* is a non-alcoholic drink produced from different substrates including rice, cassava, maize, peanut, cottonseed, banana and pumpkin by the Tapirapé Indians from Mato Grosso, Brazil. The traditional production of *cauim* involves cooking the different raw materials for approximately 2 h and then cooling them to room temperature. At this time the inoculum is added, which consists of the juice obtained from the chewing of sweet potatoes by the women of the tribe and fermented for 24 to 48 h (Schwan et al., 2007).

Schwan et al. (2007) studied the yeast diversity using a culture-dependent approach of a non-commercial *cauim* made with cassava and rice. The beverage was prepared in a traditional manner. To prepare the inoculum, the women of the tribe chew the sweet potatoes and add the saliva and the chewed sweet potatoes to the cassava and rice mixture (previously ground and cooked); in this way, the microorganisms present in the saliva act as starters. The role of saliva is to provide amylase to start the process of hydrolysing starch to fermentable sugar (Elizaquível et al., 2015). They found seven different yeast species, with *Candida tropicalis and Candida intermedia* being the most abundant. In another work, Ramos et al. (2010) studied the microbial diversity of another non-commercial *cauim* made with peanut and rice using culture-dependent (D) and -independent (I) methods. They also found that the dominant yeast genera at the end of fermentation were *Candida* (I/D) along with *Saccharomyces cerevisiae* (I/D) and *Kluyveromyces lactis* (I/D). The authors point out that the yeasts present in *cauim* fermentation would increase its nutritional value since they would be involved in starch degradation, flavour production, rising of vitamin B levels and free amino acid production (Schwan et al., 2007).

Ramos et al. (2010) also studied the bacterial diversity and found that *Lactiplantibacillus plantarum* (I/D) and *Limosilactobacillus fermentum* (I/D) dominated in the final stages of *cauim* fermentation.

#### Tarubá

**Figure 2:**
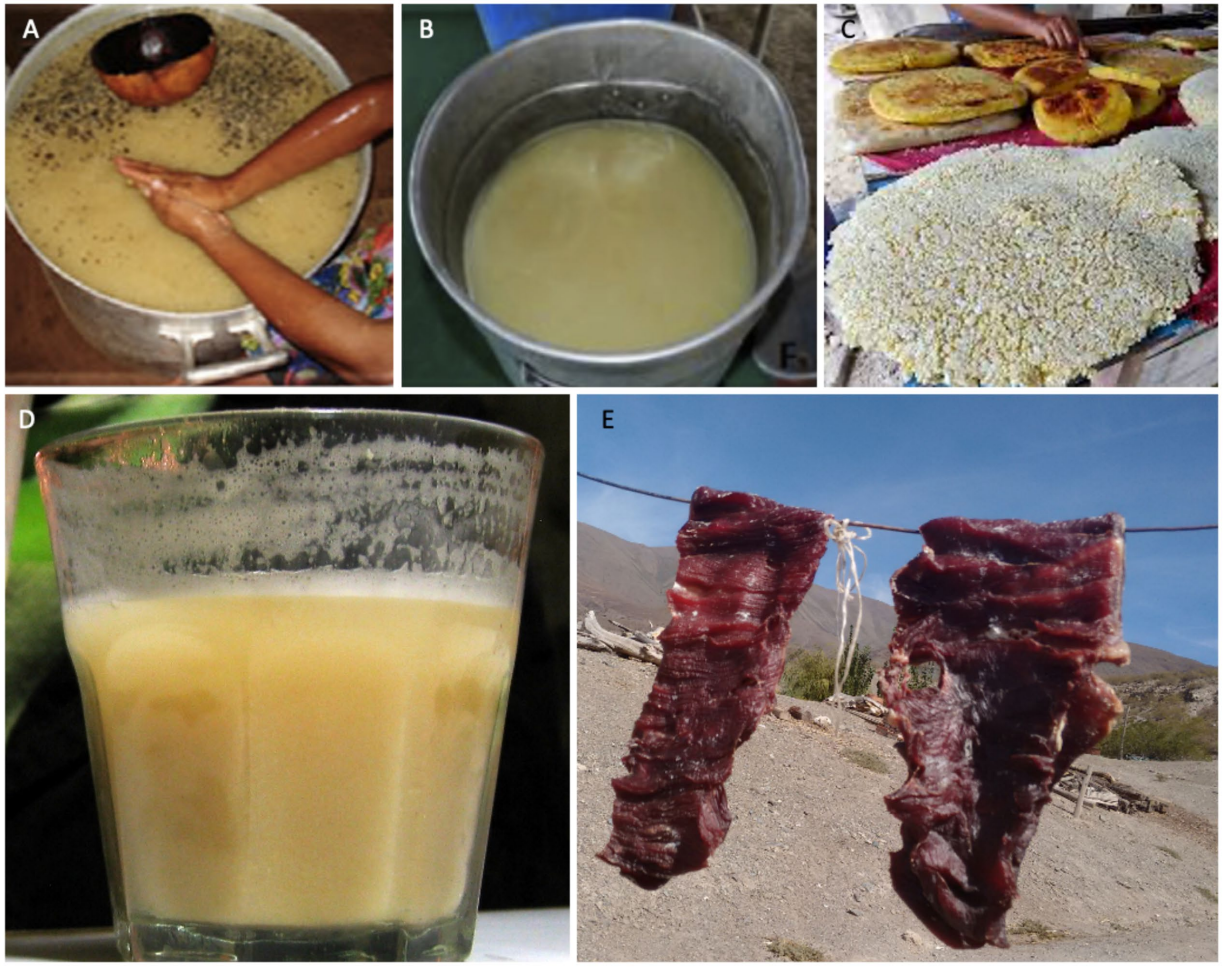
Fermented foods **(A)** *Caxiri* (Miguel et al., 2015), **(B)** *Taruba* (Ramos et al., 2015), **(C)** *Beijus* (Google, license-free), **(D)** Maize *chicha* (Google, license-free), and **(E)** Llama *charqui* (Google, License free).

*Tarubá* is a beverage that is distinctive since a solid substrate is used for fermentation. The cassava roots are cleaned, crushed, pressed, sieved and toasted for 30 min, placed in a wood receptacle (*gareira*) and covered with *Trema micrantha* (L.) Blume and *Musa* spp. (banana) leaves, and allowed to ferment for 12 days. To obtain the final beverage, the fermented cassava is diluted in water and filtered (Figure 2; Ramos et al., 2015). Examination of the microbial composition of this traditionally made non-commercial *tarubá* by culture-dependent and independent methods (PCR-DGGE; Ramos et al., 2015) shows that the dominant yeast throughout the fermentation process and in the final beverage was *Torulaspora delbrueckii*. With respect to bacterial taxonomy the authors found that at the end of the fermentation and in the final product that the dominant species were *L. plantarum, Leuconostoc mesenteroides and Bacillus subtilis*. It was suggested by the authors of this study that interactions between these microbes were responsible for flavour development due to the protease activity and volatile compound production.

#### Yakupa

Another fermented drink, produced by the Jaruma people, an indigenous population of the Mato Grosso, Brazil, is *yakupa*, a yellowish, slightly acidic and bitter beverage. It is a non-alcoholic beverage drank by the entire population daily (Lima, 2006). The preparation begins by letting cassava soak in water for 2 or 3 days, producing a pre-fermentation of the substrate (puba). The puba is dried in the sun and crushed, water is added and the mixture is sifted and cooked adding grated sweet potato. The drink can be consumed immediately or it can be left to ferment for 24 to 48 h (Freire et al., 2014).

Freire et al. (2014) studied the microbiological dynamics of non-commercial yakupa fermented over 60 h, produced by the local Amerindian Yudjá–Pakaya tribe, which is located in the Xingu Indigenous Park (Mato Grosso, Brazil) by culture-dependent and -independent (PCR-DGGE) methods. Throughout the entire fermentation process the authors found that *L. fermentum* and *S. cerevisiae* were the dominant species using both methodologies.

#### Calugi

Another non-alcoholic fermented beverage is *calugi*, which is produced by Javaé Brazilian Indians (Tocantins, Brazil) using different substrates such as rice, cassava and maize (Miguel et al., 2012). Miguel et al. (2012) characterised the microbiological diversity of non-commercial *calugi* produced from rice and maize using the mastication juice of sweet potato as an inoculum. The maize (previously peeled) and rice were cooked together for 2 h. Once the mixture had cooled, the inoculum was added and allowed to ferment at room temperature (30°C approx.) for 48 h.

Culture-independent analysis (PCR-DGGE) showed that *Streptococcus salivarius* was the most abundant bacteria and was present throughout the fermentation. *S. cerevisiae* was present during almost the entire process, and was the only yeast detected at 46 h of fermentation (Miguel et al., 2012).

#### Cottonseed and rice beverage

The Amerindian TapirapéeTapi’itãwa tribe from Mato Grosso, Brazil prepares cottonseed and rice fermented beverages for local consumption. Ramos et al. (2011) studied the microbial diversity using both culture-dependent and -independent (DGGE-PCR) methods. The cottonseed and rice were soaked in water and crushed. After that, water was added and cooked for 30 min, and then left to ferment in an open vessel for 48 h at room temperature (approximately 30°C). The authors (Ramos et al., 2011) found that the most abundant genera in the beverage were LAB (57%), mainly *L. plantarum* and *Lentilactobacillus* spp. at the end of fermentation. The yeast species found in the final product were *Candida parapsilosis and Clavispora lusitaniae*. LAB were the principal microorganisms present at the end of the fermentation process.

### Alcoholic beverages

#### Champu

*Champu* is a fermented alcoholic beverage with a sweet, sour taste consumed in the rural and urban areas of Colombia. The ingredients can include cereals (wheat, rye and maize), pineapple, or “lulo” (*Solanum quitoense* Lam.), “panela” (sugar cane tablets) syrup, clove, cinnamon and orange tree leaves. The first step in the preparation of *champu* is to soften maize grains by boiling them for several hours, before adding the other ingredients. These are then left to ferment for 24 to 48 h at a temperature of 12–15°C. The resulting alcoholic content in the beverage is between 2.5% and 4.2% with a pH between 3.5 and 4 (Osorio-Cadavid et al., 2008).

Osorio-Cadavid et al. (2008) studied the yeast diversity present in 20 different commercial *champu* through a culture-dependent analysis (Table 1). They found 8 different species of yeast, where *Issatchenkia orientalis, S. cerevisiae* and *Pichia fermentans* were the most abundant species. According to the authors (Osorio-Cadavid et al., 2008), the diversity of yeasts present may be a reflection of the raw material used. These species were also found in different fermented beverages made using the same substrates (Sefa-Dedeh et al., 1999; Wacher et al., 2000; Botes et al., 2007), in sour cassava (Lacerda et al., 2005) and coffee fermentations (Masoud et al., 2004).

**Table 1:** Microorganisms present in artisanal fermented foods from South America.

Fermented food	Country	Substrate	Microorganism	References
**Fermented beverages**				
*Champu*	Colombia	Maize, sugar cane molasses, clove, cinnamon, orange tree, pulp fruit	*I. orientalis, S. cerevisiae, P. fermentans, G. geotrichum, P. kluyveri var. kluyveri, T. delbrueckii, Hanseniaspora spp.* *and P. fermentans*	Osorio-Cadavid et al., 2008
*Cauim*	Brazil	Peanut, rice, sweet potato	*L. plantarum, L. fermentum, L. paracasei, L. brevis, Leu. pseudomesenteroides, Bacillus* *sp., P. guilliermondii, K. lactis, Candida* *sp., R. toruloides, C. albicans, and S. cerevisiae*	Ramos et al., 2010
	Brazil	Cassava, rice, sweet potato	*C. tropicalis, P. guilliermondii, C. intermedia, C. parapsilosis, T. asahii, S. cerevisiae and E. dermatitis*	Schwan et al., 2007
*Tarubá*	Brazil	Cassava	*T. delbrueckii, P. exigua, H. uvarum, C. rugosa, C. tropicalis, P. kudriavzevii, W. anomalus, C. ethanolica and P. manshurica, R. mucilaginosa, L. plantarum, L. brevis, Leu. mesenteroides, Leu. Lactis, P. pentosaceus, B. subtilis, B. amyloliquefaciens, B. licheniformis, Bacillus sp., A. orientalis, C. terrae and O. intermedium*	Ramos et al., 2015
*Chicha*	Argentina	Maize, cane sugar	*Lc. mesenteroides, Lc. lactis; L. plantarum, L. brevis, F. Rossiae, W. viridescens, W. confusa, E. casseliflavus, E. faecium, E. mundtti, E. durans, E. hirae, P. acidilactici and Streptococcus*	Elizaquível et al., 2015
			*S. cerevisiae, C. parapsilosis, C. oleophila, Pichia sp., Cryptococcus, D. hansenii, G. candidum, K. lactis, M. caribbica, P. fermentans, T. domesticum and Fusarium*	Mendoza et al., 2017
	Brazil	Rice	*Propionibacterium sp., Bifidobacterium sp., Leu. lactis, L. casei, Enterobacter sp., E. coli, E. lactis, Streptomyces sp., B. subtilis, E. durans and K. pneumonia*	Puerari et al., 2015b
		Maize and cane sugar	*W. confusa, W. cibaria, K. pneumoniae, K. quasipneumoniae, B. anthracis str Ames, B. safensis, S. saprophyticus, S. capitis, E. kobei, M luteus, R. mucilaginosa, L. elongisporus, C. metapsilosis and C. bohaiensis*	Resende et al., 2018
		Bacaba fruit and sugar cane	*E. durans, E. hirae, Enterococcus spp., E. hormaechei, Enterobacter spp., Leu. lactis, P. dispersa, P. avidum, Acetobacter spp., P. caribbica, P. guilliermondii and P. caribbica*	Puerari et al., 2015a
	Ecuador	Cassava	*L. acidophilus, L. reuteri, L. delbrueckii, L. fermentum, L., Lactobacillus sp.* (Previously undescribed species)*, Acetobacter pasteurianus, Bacillus spp.,* others	Colehour et al., 2014
	Peru	Maize	*Lactobacillaceae* (*L. plantarum, L. fermentum*, and *W. cibaria*)*, Acetobacteraceae* (*A. okinawensis*)*, Leuconostocaceae, Streptococcaceae* (*S. luteciae* and *S. alactolyticus*)*, Clostridiaceae, Bacillaceae, Enterobacteriaceae, Enterococcaceae, Bifidobacteriaceae, Sphingomonadaceae* and *Ruminococcaceae*	Bassi et al., 2020
*Caxiri*	Brazil	Cassava and sweet potato	*P. acidilactici, B. subtilis, Bacillus* spp. (cereus group), *B. amyloliquefaciens, B. pumilus, B. megaterium, B. simplex, B. mannanilyticus, Lc. lactis subsp. Lactis, L. sphaericus; L. fusiformis, Sphingomonas sp., L. Fermentum, Curtobacterium* sp., *K. pneumonia, S. mutans, Enterobacter sp., R. mucilaginosa, P. membranifaciens, P. guilliermondii, C. luteolus* and *S. cerevisiae*	Santos et al., 2012
	Brazil	Cassava, maize and sweet potato	*B. subtilis, L. fermentum, L. helveticus, K. pneumonia, S. carnosus, E. coli, E. cloacae S. cerevisiae, P. kluyveri, C. tropicalis R. mucilaginosa* and *D. fabryi*	Miguel et al., 2015
*Yapuka*	Brazil	Cassava and sweet potato	*L. fermentum, L. plantarum, W. cibaria, W. confusa, S. cerevisiae* and *P. kudriavzevii*	Freire et al., 2014
*Calugi*	Brazil	Rice, maize and sweet potato	*L. plantarum, S. salivarius, S. parasanguinis, W. confusa, E. cloacae, B. cereus, Bacillus sp.,* *S. cerevisiae, P. fermentans* and *Candida* sp.	Miguel et al., 2012
Cottonseed and rice beverage	Brazil	Cottonseed and rice	*L. plantarum, L. paracasei, Lentilactobacillus spp., L. sakei, L. brevis, C. maltaromaticum, Leu. Lactis, Leu mesenteroides, Lc. Lactis, Bacillus sp.,* *B. subtilis, B. cereus, Corynebacterium, R. mucilaginosa, C. parapsilosis, C. lusitaniae* and *Candida orthopsilosis*	Ramos et al., 2011
**Meat products**				
Dry-cured sausages	Argentina	Beef, goat, lamb, llama and guanaco meat	*L. plantarum, L. sakei, S. saprophyticus, S. xylosus, S. equorum, Kocuria spp., E. faecium, E. hirae, E. casseliflavus, Leuconostoc spp., Leu. inhae, Leu. mesenteroides, Leu. lactis. W. ghanensis, W. viridescens, C. viridans, Pseudomonas spp., Psychrobacter spp., Acinetobacter spp*.,	Beserra et al., 2003; Lahti et al., 2001; Fontana et al., 2005, 2016; Cocconcelli et al., 2012
*Charqui, jerked beef, carne-de-Sol*	Brazil	Beef, pork, goat, lamb, llama and guanaco meat	*Pediococcus sp., Micrococcus sp., Lactobacillus sp., Streptococcus sp. and Lactococcus sp., Staphylococcus sp., S. xylosus, S. pasteuri, S. warneri, S. saprophyticus, S. carnosus, Lc. lactis, M. roseus, H. cutirrubrum, H. salinarum, S. littoralis, S. marcescens*	Shimokomaki et al., 2016; Pinto et al., 2002; Biscola et al., 2013, 2014; Aymerich et al., 2003; Cocolin et al., 2004; Albano et al., 2008; Tu et al., 2010; Fadda and Vignolo, 2014; Penna et al., 2017
**Starchy products**				
*Tocosh*	Peru	Potato, papalisa, oca or arracacha	*L. sakei, L. casei, L. farciminis, L. brevis, L. fermentum, Leu. mesenteroides, Clostridium sp., Zymophilus sp., Prevotella sp., moulds and yeasts*	Jiménez et al., 2018; Yépez et al., 2017
*Beijus* and *Tiquira*	Brazil	Cassava	*Paecilomyces sp., A. niger, Penicillium, Rhizopus sp., N. crassa, Manila sitophila, Saccharomyces spp.*	Park et al., 1982; Chuzel and Cereda, 1995; Penna et al., 2017
*Puba* flour	Brazil	Cassava	*Lactobacillus and Leuconostoc sp., L. fermentum, L. delbrueckii, L. plantarum* group*, L. perolans, L. brevis, Enterobacteriaceae, Corynebacterium sp., Bacillus sp., Clostridium sp.,* yeasts, moulds.	Lacerda et al., 2005; Padonou et al., 2010; Crispim et al., 2013; Anyogu et al., 2014; Fayemi and Ojokoh, 2014; Penna et al., 2017
*Masa agria*	Colombia	Maize	*Lactobacillus* (*Lb. gallinarum, Lb. fermentum, Lb. plantarum, Lb. pontis, Lb. panis, Lb. coleohominis*, and *Lactobacillus spp.*), *Acetobacter* (*A. cibinongensis, A. fabarum, A. lovaniensis*, *A. orientalis* and *Acetobacter spp.*), *W. fabaria, Pediococcus, Sphingobium*, *Comamonas, Enterobacter, Escherichia, Serratia*, *Acinetobacter, Thiothrix. Pseudomonas* and *Gemma*	Chaves-Lopez et al., 2016
**Dairy products**				
Cheese	Brazil	Cows milk	*E. faecalis, L. acidipiscis, L. brevis, L. buchneri, L. parabuchneri, L. paracasei, L. casei, L. plantarum, L. rhamnosus, Lc. Garvieae, W. paramesenteroides, Lc. lactis, L. curvatus, L. fermentum, L. delbrueckii, S. thermophilus, P. acidilactici, L. mesenteroides, K. kristinae, E. coli, Staphylococcus spp., S. salivarius, S. aureus, Streptococcus sp., L. plantarum, Lactobacillus sp., Lactococcus sp., Weissella sp., Klebsiella oxytoca, Serratia marcescens, Citrobacter freundii and Streptococcus macedonicus*	Perin et al., 2017; Arcuri et al., 2013; Sant’Anna et al., 2019; Camargo et al., 2021
	Argentina	Ewes milk	*L. plantarum, L. acidophilus, L. casei*	Medina et al., 2001
	Argentina	Goats milk	*L. plantarum*, *L. rhamnosus*, *L. delbrueckii* *subsp. Bulgaricus*, *L. fermentum*, *E. faecium*, *P. pentosaceus, L, casei, L. paracasei* and *Lc. Lactis*	Oliszewski et al., 2007; Rivas et al., 2014
	Brazil	Ewes milk	*L. plantarum and L. rhamnosus*	Nespolo and Brandelli, 2010
*Suero costeño*	Colombia	Cow’s milk	*L. plantarum, L. paracasei, L. acidophilus, L. brevis, L. delbrueckii, L. pentosus, L. rhamnosus, Lc. Lactis, Leu. Lactis, Leu. Mesenteroides, Enterobacteria sp., C. parapsilosis, P. kudriavzevii, S. cerevisiae, E. faecium, S. infantarius, S. salivarius, Streptococcus, Lactobacillus, Aeromonas, Lactococcus, Acetobacter, Bifidobacterium, Leuconostoc, Staphylococcus, Acetobacter, Enterobacter, Pantoea, Klebsiella and Escherichia-Shigella*	Cueto et al., 2007; Motato et al., 2017
*Kumis*	Colombia	Cow’s milk	*L. plantarum, T. delbrueckii, G. geotrichum, P. kudriavzevii, K. marxianus, E. faecalis, E. faecium, E. serioliocida, V. penaei, C. tropicalis, C. glabrata, C. pararugosa, C lusitaniae, K. unispora, R. mucilaginosa, T. asahii, T. insectorum, S. cerevisiae and W. pjiperi*	Chaves-López et al., 2011, 2012, 2014b

#### Chicha

*Chicha* is a traditional homemade fermented beverage produced by the native populations in South America (Figure 2). This alcoholic beverage (2%–12% v/v) is mainly made from maize but other raw materials such as rice, cassava, peanut, fruit, or carob can also be used. It is consumed during religious and agricultural festivities as well as during family and social events (Lorence-Quiñones et al., 1999; Delibes and Barragan Villena, 2008). In Peru, *chicha* is prepared in a traditional way by the indigenous population using local maize varieties, it is called *chicha de jora* and has a low alcoholic content (1%–3%; Delibes and Barragan Villena, 2008). Although the traditional fermentation process relied on saliva as an inoculum, this has increasingly changed to employ approaches to replace the amylase present in the saliva (Lorence-Quiñones et al., 1999).

##### Commercial *chichas*

Bassi et al. (2020) studied the microbial population of 27 *chichas* made with different recipes and raw materials such as maize, barley, wheat, quinoa, fava beans, maca, sugar, panela, fruits (orange, banana, apple) and spices (cinnamon, cloves). The *chichas* were collected from 14 different “Chicherias” from Peru and analysed using HTS (high throughput sequencing). Eleven different bacterial families were found, with *Lactobacillaceae* being the most abundant in most *chichas*, although *Acetobacteraceae*, *Leuconostocaceae* and *Streptococcaceae* dominated in three, one and one *chicha* samples, respectively, representing 50% of the total population in each case (Bassi et al., 2020).

##### Maize *chicha*

In Argentina, maize *chicha* is prepared in the northwest, in the Andean region. Elizaquível et al. (2015) studied the microbial biodiversity of non-commercial *chichas* produced in two different towns (Maimara and Tumbaya) in the province of Jujuy comparing culture-dependent and -independent methods (HTS). The production of both *chichas* involved several phases with a two-step fermentation process (Elizaquível et al., 2015; Mendoza et al., 2017). In Maimara *chicha* (*chicha* M) Elizaquível et al. (2015) found that the most abundant species of bacteria were *Leuconostoc lactis* (I/D), *L. plantarum* (I/D), *Weissella viridescens* (I) and *Furfurilactobacillus rossiae* (I). In the *chicha* from Tumbaya (*chicha* T) they found *E. faecium* (I/D), *Leu. mesenteroides* (I/D), *Enterococcus hirae* (I) and *Weissella confusa* (I).

Regarding the composition of yeasts present in these beverages, Mendoza et al. (2017) found through a culture-based approach and with HTS method that *S. cerevisiae* (I/D) was the most abundant yeast present in both *chicha* M (72%) and *chicha* T (31%). Among the filamentous fungi, the genus *Fusarium* (I) was the most abundant (Mendoza et al., 2017).

Resende et al. (2018) studied the microbial community present in maize and sugar cane *chicha* prepared by the indigenous people of Guarani-Kaiowá ethnicity at Jaguapiru village (Dourados, Mato Grosso do Sul, Brazil) using identification of isolates by protein profiling using MALDI-TOF and confirmation by partial 16S sequencing. They found that *W. confusa* was the most abundant bacteria and *Candida bohaiensis* the most abundant yeast (Resende et al., 2018).

##### Rice *chicha*

The indigenous Umutina tribe from Barra do Bugres (Mato Grosso, Brazil), also prepare a beverage called *chicha* but use rice instead maize or, more specifically, using chewed rice as an inoculum (Puerari et al., 2015b). The most abundant bacteria found in rice *chicha* by culture-dependent and independent (PCR-DGGE) methods was *Lacticaseibacillus casei.*

##### Bacaba *chicha*

*Bacaba chicha* is another *chicha* made by the Umutina tribe and is prepared with bacaba fruit (*Oenocarpus bacaba*). The fruit is soaked in boiling water, crushed and the skin and seed removed, sugar cane is added and the beverage can be drunk immediately or left to ferment for a few hours (Puerari et al., 2015a). A combination of culture-dependent and independent (PCR DGGE) approaches showed that the most abundant bacteria and yeast present in *bacaba chicha* were *Enterococcus durans* and *Pichia caribbica*, respectively

##### Cassava *chicha*

Indigenous Amazonian groups also brew *chicha* from sweet cassava. *Chicha* from cassava, also called *masato*, is typically a low alcohol beverage, with a milky consistency and somewhat sour flavour. Amongst Shuar aborigines, c*hicha* is typically prepared over a 2–3 days period. First, the cassava roots are peeled, washed and boiled until soft. Water is then drained off and the root mashed with a dedicated pestle, while the brewer masticates pieces of the cassava and periodically spits into the mash. The finished mash is placed in a designated vessel to ferment for 1–3 days, depending on preference for sweet to sour *Chicha* (Penna et al., 2017).

Culture-based studies on samples from cassava fermented beverages highlight the dominance of LAB (Santos et al., 2012). In another study using HTS approaches, Colehour et al. (2014) described the microbial composition of cassava *chicha* from different Shuar villages and households in Ecuador. Although *Bacillus* dominated (64%) in the unfermented material, *Lactobacillus* and *Acetobacter* collectively accounted for 71% of the sequences in all fermented samples. *L. acidophilus* represented 51% of the identified lactobacilli and *Acetobacter pasteurianus* were the next most abundant species. Notably, bacterial communities in *chicha* were significantly different between villages, but not significantly different across households within a village. It was suggested that oral microbiome swapping occurs but that, rather than resulting from the chewing/spitting processes during preparation of the beverage is actually more likely to happen during consumption than during production, when the drinking cups, *pilchis*, are passed around a social gathering and dipped repeatedly into the fermentation vessel after each person takes a drink. The authors considered that, over many generations, this traditional process of spontaneous fermentation can be considered a process of microbial domestication if microbe assemblages are consistently distinct (Colehour et al., 2014).

#### Caxiri

*Caxiri* is a spontaneously fermented alcoholic beverage manufactured from cassava by the Yudja women of the Pakaya tribe from Brazil, consumed particularly in ceremonies and festivities (Lima, 2006). To prepare *caxiri*, cassava roots are fermented for 2 days in running water to soften the skin. Then, the cassava tubers are peeled, cut into small pieces, pressed, grated and roasted. The resulting flour is subsequently mixed with water and thereafter placed in barrels to initiate the fermentative process, which usually takes 24 to 48 h (Figure 2). During this fermentation, besides the degradation of cyanogenic compounds and formation of aromatic substances, softening of the roots occurs caused mainly by LAB (Kimaryo et al., 2000). Generally, *caxiri* is consumed up to 120 h after preparation (Santos et al., 2012).

The study of the microbiological composition of non-commercial *caxiri* prepared with casava and sweet potato with culture-dependent methods (Santos et al., 2012) revealed that the *Bacillus* genus (*subtilis, cereus, amyloliquefaciens* among others) were dominant since the beginning to the end of the fermentation processes. Regarding to the LAB, *Pediococcus acidilactici* was the only specie found at the end of the fermentation. At the beginning of fermentation, different specie of yeast were found, being with *S. cerevisiae* being the dominant species through all the process.

Other authors (Miguel et al., 2015) studied the microbial diversity present in a non-commercial *caxiri* prepared with cassava, maize and sweet potatoes. They found that *S. cerevisiae* was also the dominant yeast during the entire process, but LAB were the dominant bacteria, with *L. fermentum* and *Lactobacillus helveticus* being the dominant species, unlike what was found by Santos et al. (2012) where the *Bacillus* genus was dominant. For this study, the authors used culture-dependent methods and DGGE and PCR analysis.

The production of artisanal fermented beverages involves a consortium of microorganisms, mainly bacteria and yeasts. Throughout the fermentation process, the type of predominant microorganism may vary (Blandino et al., 2003).

Through the fermentation process, yeasts participate in the degradation of starch, in the development of flavour, increasing vitamin B levels and free amino acid production, with the consequent increase in nutritional value (Schwan et al., 2007). *Saccharomyces* cerevisiae to *S. cerevisiae* was the dominant yeast in several fermented beverages (*cauim, yakupa, calugi, champu and chicha*) This yeast is mainly associated with ethanol, CO_2_ production and other aromatic components, such as esters, organic acids and carbonyl compounds (Janssens et al., 1992; Torner et al., 1992).

LAB are the main bacteria genus found in most fermented beverages. They have an important role due to their ability to metabolize different carbon sources (McDonald et al., 1990). The growth of these bacteria could favour the growth of yeasts due to the acid production. Likewise, the production of vitamins and nitrogenous compounds by yeasts stimulates the growth of LAB (Nout and Sarkar, 1999). Another essential role of LAB is the production of antimicrobials that, in addition to acid production, create unfavourable conditions for the growth of pathogens, toxigenic and spoilage organisms, improving the safety and stability of the final product (Miguel et al., 2012).

Bacteria belonging to the genus *Bacillus* were found in different fermented beverages (*calugi, caxiri, cotton seed rice beverage, chicha and taruba*). They may be involved in the secretion of various enzymes such as amylases, lipases, cellulases and proteases, which are essential in the fermentation process (Rusul and Yaacob, 1995; Amoa-Awua et al., 1997; Obilie et al., 2004).

Co-metabolism between the different microorganisms improves the characteristics of the final products through flavour development, improvement of food safety, reduction in cyanogen glucoside levels, among other activities (Damiani et al., 1996; Obilie et al., 2004; Kamda et al., 2015).

## Fermented foods

### Dairy products

While globally milk is a commonly used substrate for fermented food production, its use in South American countries is generally lower than, for example, European countries (Tovar and Herrera, 2017; Tamang et al., 2020). Although data is not available on fermented dairy products alone, consumers in South America generally spend less than half on dairy products overall compared to their European counterparts. However, a number of fermented dairy products are produced in South American countries including *kumis*, cheeses and *suero costeño*, with cow’s milk being the most common substrate of dairy fermentations. The highly perishable nature of dairy products makes the use of fermentation desirable for dairy products to extend shelf life while also adding unique flavour profiles and, potentially, health-promoting components.

#### Kumis

*Kumis* (or *Koumiss*) is a fermented dairy beverage originated in Central Asia and commonly produced and consumed today in Russia. However, here our focus is on Colombian *kumis*. While traditionally *kumis* is produced with mare’s milk, the fermented beverage produced in Colombia is based on raw whole cow’s milk (Chaves-López et al., 2011) This beverage is fermented in rural and urban areas of South West Colombia with production slightly different from other variations produced in other countries. It is very similar to kefir, a sour and fizzy milk-based fermented drink (Bourrie et al., 2016), except that a liquid starter culture is used by back-slopping from the previous ferment. The milk is allowed to ferment for 2–3 days, with sugar cane and cinnamon added, before serving as a low alcohol (1%–2%), creamy, sour and sparkling beverage (Chaves-López et al., 2011).

Research into Colombian *kumis* is limited, with much of the research to date originating from within one group, and the full microbial content of *kumis* from Colombia not yet elucidated. *Kumis* of other origins have been identified as having lactobacilli and yeasts as the main microbial components (Samat Kozhakhmetov et al., 2014). Analysis from Chaves-López et al. (2011); Chaves-López et al., (2012, 2014b) has investigated the composition of Colombian *kumis*, with a particular focus on the potential angiotensin I converting enzyme (ACE) inhibitory activity of the microbes identified. These investigations highlighted similar trends as was seen with *kumis* from other origins within commercially produced *kumis*, with LAB and yeast dominating the populations present with counts of 7.05–9.59 log CFU g^−1^ and 6.26–8.65 log CGU g^−1^, respectively. In addition to the yeast and LAB populations from *kumis*, Chaves-López et al. (2011) have identified enterococci as part of the main microbial composition of Colombian *kumis* with counts of 4.29–8.3 log CFU g^−1^. Although the yeast and enterococcal populations of Colombian *kumis* were further characterised, a thorough investigation of LAB from the *kumis* was not completed (Chaves-López et al., 2014a). Amongst yeast species identified from Colombian *kumis*, *Galactomyces geotrichum* was most commonly isolated (22.58% of total yeasts isolated; Chaves-López et al., 2012). Several other yeast species were isolated from Colombian *kumis* (Table 1) with 21 different RAPD-PCR profiles identified from the 93 isolates (Chaves-López et al., 2012). Enterococci were also investigated in further detail from Colombian *kumis* with 72 isolates identified, with *Enterococcus faecalis* being the dominant species (Chaves-López et al., 2011).

From ACE1 inhibitory investigations on isolated strains from *kumis*, proteolytic activity was identified for several yeasts and enterococci investigated with these activities potentially contributing to the flavour profiles developed in the *kumis*. This activity may contribute to health benefits following consumption, although this was not fully determined through these investigations (Chaves-López et al., 2011, 2012). These strains could be utilised in the production of *kumis*, although some are likely better suited to such industrial processes than others (Chaves-López et al., 2014a).

#### Cheese

As it is observed in many countries where cheese production occurs, there are a wide variety of cheeses available across South America, varying in their ingredients, production methods and fermentation processes. Despite the large variety of cheeses available within South America, only a small proportion of these have been investigated in relation to their microbial composition and only those are highlighted here. Although several immigrant methods are used to produce cheese in South America (with the exception of the indigenous *Minas* cheese) as these cheeses have been produced over many generations and slight variations to methods, they could now be considered indigenous.

*Minas cheese* is a semi-soft cheese produced in Minas Gerais state, Brazil with varieties named according to geographical region. Generally, this cheese is produced from raw cow’s milk, although occasionally pasteurised milks may be used, and ripened for up to 60 days, with ripening length varying by producer. The starter culture used in this cheese production (called pingo) is from the whey of the previous cheese production (Arcuri et al., 2013; Perin et al., 2017; Sant’Anna et al., 2019). Large variation has been identified within the microbial composition of *Minas cheeses*, with variation not only evident between geographical regions (Arcuri et al., 2013) but also between individual production farms (Perin et al., 2017). This variation is likely due to the combined impact of the use of a raw milk starter, which unsurprisingly varies between farms (Perin et al., 2017), and the use of the “back-slopping” approach through the use of the whey from the previous production. Although specific details are not given on production methodologies utilised for each sample it is possible that some variation may be introduced here also.

Studies of commercially produced *Minas cheeses* have also identified some variation between studies with Perin et al. (2017) identifying *Lactobacillus* species as predominant members, with variation between the species based on geographical origin, while Sant’Anna et al. (2019) identified *Streptococcus* as the genus to which most OTUs identified belonged. These differences may be due to factors such as ripening time variation between studies, which would strongly influence microbial composition, although total ripening time of cheeses investigated in the case of Perin et al. (2017) are not given. The variation observed here in dominant members would have an influence on the properties and characteristics of these cheeses (Ahmed et al., 2021), although this has not been specifically investigated in *Minas cheeses.* In addition, Sant’Anna et al. (2019) identified a variance in microbial species over time with *Streptococcaceae* and *Planococcaceae* present at all stages of the ripening process while *Lactobacillaceae* and *Leuconostocaceae* appear in the later stages of the ripening. Camargo et al. (2021) studied commercial *Minas Gerais* cheese from *Entre Sierras* region. Samples from 4 different producer were analysed, in the same way as Perin et al. (2017), and the authors found that LAB were the predominant bacteria amongst all the samples. *Lactococcus* was the main genus identified with*. Lc. Lactis* being present in all samples (Camargo et al., 2021). Microbial succession has also been investigated in other cheeses (Fuka et al., 2013; O’Sullivan et al., 2015), with the variation in species present influencing end products of biochemical events during ripening ultimately dictating the properties of the final cheese (McSweeney, 2004). Overall, a variety of factors seem to have an influence over the microbial composition of *Minas cheese*. The variance in the members identified may be associated with the variance in flavour and characteristics of these cheeses between regions.

Argentinian-produced cheeses are predominantly produced using milks other than cow’s milk, especially goat and sheep, using immigrant methods commonly used in cheese production around the world. These semi-hard cheeses tend to be produced from the raw milks with little standardisation in the process involved (Medina et al., 2001; Oliszewski et al., 2007). The investigation of one such commercial semi-hard ewe’s milk cheese produced without a starter culture and which was ripened at 20°C for 30 days identified the dominance of *L. plantarum*, making up 93% of isolates (Medina et al., 2001). The *L. plantarum* were associated with important contributions to the ripening stages and flavour development, being involved with a number of enzymatic activities which may be responsible for the characteristic flavour of this cheese (Medina et al., 2001). Examination of an ewe’s milk cheese commercially produced in Brazil through similar processes (i.e. raw milk and no starter) identified a high proportion of LAB throughout the ripening period (3 months) from 6.0 CFUg^−1^ to 6.7 CFUg^−1^, with *L. plantarum* also identified here, although full characterisation of all strains present was not completed (Nespolo and Brandelli, 2010). Investigations of enzymatic activities of isolates from this cheese highlighted *Lc. Lactis* spp. *lactis* and *L. plantarum* as important contributors to lysis activities contributing to flavour development.

Goat’s milk in Argentina is generally used for the production of fermented foods rather than for direct consumption. Investigations of raw goat’s milk cheeses have yielded varying results between studies, which is not surprising given the use of raw milk and the absence of starters, similar to the process of the ewe’s milk cheeses. Although the diversity of species appeared to be higher in goat’s milk rather than that in ewe’s milk cheeses, this may be as a consequence of the greater number of studies and the variation in methods used to identify species with respect to the former. Across goat’s milk studies the most dominant species also varied, being either *L. plantarum, L. fermentum,* or *Lacticaseibacillus rhamnosus* depending on the study (Oliszewski et al., 2007; Rivas et al., 2014). Together these studies have identified *Lactobacillus* and *Enterococcus* species as the major LAB in goats milk cheeses that contribute to the ripening processes and flavour development through several enzymatic activities (Oliszewski et al., 2007; Rivas et al., 2014).

Several efforts have been made to attempt to standardise the production of ewe’s and goat’s milk cheeses in Argentina (Oliszewski et al., 2008; Bergamini et al., 2010). Both mixed and single strain starters have been investigated including strains of *Streptococcus thermophilus*, *Lactobacillus helveticus*, *Lactobacillus bulgaricus*, *L. plantarum*, *L. rhamnosus* and *L. casei*. As expected, different outcomes were apparent depending on the strain(s) used, however there were broadly beneficial effects with respect to the prevention of spoilage and pathogenic bacteria (Oliszewski et al., 2008; Bergamini et al., 2010).

Overall, despite the large variety of cheeses available within South America only a small proportion of these have been investigated. Variation in microbial composition could be observed between products originating from different milks (cow’s, goat’s, or ewe’s), which is to be somewhat expected, although geographical region and production method variation could also be at play here. Cheeses investigated across studies are predominantly artisanally produced, with little consistency in methodologies (for example ripening times vary, when outlined, from 10 to 90 days), and so as such the microbial diversity observed here in species identified is expected, although some species including *L. plantarum* were found to be commonly identified within many cheeses which is in line with investigations of cheeses globally (Xanthopoulos et al., 2000; Belicová et al., 2013; Fernandes et al., 2017). This species is well characterised with respect to its probiotic potential and importance in food safety, making it an important member of these cheeses (Dinev et al., 2018; Khalil et al., 2022).

#### Suero costeño

*Suero costeño* is an indigenous spontaneously fermented cow’s milk product produced in the Colombian Caribbean coast, with primary production occurring in rural areas in the departments of Antioquia, Bolívar., Córdoba and Sucre. This sour cream-like product is traditionally produced using raw cow’s milk with the final product having a slightly lumpy texture with an acidic and salty taste. Raw milk is traditionally added to calabashes (container made from a gourd), although plastic vessels can also be used, with the continuous use of the same vessels and the environment seeding the fermentation (Cueto et al., 2007; Motato et al., 2017; Tirado et al., 2017; Castillo-Solano et al., 2018). Although not typical in *suero costeño* production, some producers also utilize back-slopping (Motato et al., 2017). Fermentation occurs over a period of 12 h to 3 days, depending on the desired viscosity, at an ambient temperature (~30°C) and at a high humidity. The resultant fermented product is in 2 phases with the liquid whey removed and the creamier product retained and salted at 1–3% for consumption as *suero costeño*, most commonly as a dressing (Cueto et al., 2007; Motato et al., 2017; Tirado et al., 2017; Castillo-Solano et al., 2018). This product is most commonly artisinally produced as any industrial production, which commonly uses pasteurised milk, is not widely accepted due to the variance in taste and texture (Tirado et al., 2017).

Only a few studies have investigated the microbiological composition of *suero costeño* with variation presumably due to a combination of the impact of the raw milk used, spontaneous fermentation and short fermentation time. Counts of bacteria and yeasts varying from 9 × 10^1^ to 1 × 10^8^ CFU ml^−1^, with lower counts identified with one producer who utilised back-slopping (Cueto et al., 2007; Motato et al., 2017). Lower counts of coliforms and *Listeria* counts were particularly evident for cheeses made using back-slopping (Motato et al., 2017), although overall these bacteria were low across samples (Cueto et al., 2007; Motato et al., 2017). Although back-slopping is not routinely utilised for the production of *suero costeño*, this potentially indicates a possible benefit in its use to control undesirable microbial growth during fermentation.

*Lactobacillus*, *Lactococcus*, *Leuconostoc*, *Streptococcus*, *Aeromonas* and *Acetobacter* were the dominant genera within commercially produced *suero costeño* samples, with a variance observed in dominant members identified between producers, regions and even batches (Cueto et al., 2007; Motato et al., 2017). Other taxa were also observed at lower abundances or rarely amongst samples investigated (Table 1), with some variance also depending on methodologies used (Cueto et al., 2007; Motato et al., 2017). Samples from different manufacturers investigated by Motato et al. (2017) from within the same area (Planeta Rica) were more similar to each other than the samples produced in the other area investigated (Caucasia). Although LAB were identified as the predominant members of the final fermentation when both UHT and raw milk were used, the microbial succession varied with aerobic mesophilic bacteria or yeasts dominating at earlier time points with UHT and raw milk, respectively (Cueto et al., 2007). Although the final microbial populations were similar, the variance during the fermentations between inputs may result in a variance in taste and texture of the final product.

The variance of these inputs may offer a potential to alter *suero costeño* to consumers’ preferences through the variance of the starter substrate. Another way in which this might be achieved could be through the addition of substrates at the beginning of the fermentation. For instance, the addition of whey to a goat’s milk *suero costeño* improved overall texture of the product, offering a potential alternative to the production with cow’s milk (Tirado et al., 2017). More research may be needed into this area for the understanding of the role of each of the members present within *suero costeño* and how the alteration of substrate may vary the final product and consumer perception.

Although dairy consumption is comparatively lower in South American countries there are still several fermented dairy products produced and consumed. Cow’s milk, as is the case in many countries, is the main substrate used in these fermentations, although certain regions favor the use of ewes or goats’ milks. These fermented dairy products share several common microbial members with *L. plantarum*, *L. paracasei*, *Lc. lactis* and *L. casei* commonly found amongst the majority of fermented dairy products from South America investigated. Although methodologies vary between studies reducing the potential for accurate comparisons, some geographical patterns could also be noted, for example with the presence of *Pichia kudriavzevii* in Colombian cow’s milk products and *L. buchneri* and *L. parabuchneri* in Brazilian cow’s milk products. Overall, an abundance of further research is needed to more thoroughly cover the breadth of diary fermented foods available in South America fully.

### Meat products

A diverse range of fermented meat products are traditionally produced in South America, including sausages, *charqui*, jerked beef and *carne-de-sol*. In addition to more general fermentation benefits, meat fermentation add value to cuts with low or no commercial value (Beserra et al., 2003). In many cases, meat fermentation is combined with a drying process. This combination further enhances food safety, compared to fermentation alone, reducing the numbers of *Escherichia coli* O157:H7 and *Listeria monocytogenes*. In addition to reducing water availability through drying, protection is provided through lactic acid and bacteriocin production by the LAB naturally present in the meat batter or added as starter cultures (Lahti et al., 2001).

Previous microbial diversity studies on fermented meat products, as indicated for fermented sausages, found that LAB (mainly Lactobacilli) and coagulase-negative cocci (mainly *Staphylococcus* spp.), which prevail in these products, have a determinant role in development of their peculiar sensorial characteristics (Aymerich et al., 2003; Cocolin et al., 2004; Albano et al., 2008; Tu et al., 2010; Fadda and Vignolo, 2014).

#### Dry-cured sausages

Salamis and salami-type sausages are the most common South American fermented meat products. Dry-cured sausages have traditionally been produced by a combined process of fermentation and no-heat drying treatments using different types of meat with some variations observed across regions or countries. Goat and lamb meat are used in some countries such as Brazil and Argentina. Llama (*Lama glama*) and guanaco (*Lama paco*) meat have a high protein value and low levels of fat and cholesterol, and are widely used in the Andean highlands in fermented meat products (Cristofanelli et al., 2004; Mamani-Linares and Gallo, 2013). Most fermented meat products from South America are artisanally manufactured. In Argentina, the manufacture of dry-fermented sausages (“salame”) in the main production areas is 10% through industrial processes and 90% through local artisanal techniques (Canel et al., 2013).

LAB (mainly lactobacilli) and Gram-positive coagulase-negative cocci (*Kocuria* and *Staphylococcus*) are the main bacterial groups present during dry-cured sausage fermentation. LAB rapidly lower the matrix pH and secrete bacteriocins which improves the hygienic safety preventing the growth of spoilage and pathogenic microorganisms. This also contributes to texture and color development. *Kocuria* and *Staphylococcus* also influence the typical cured red color through the reduction of nitrate to nitrite (Toldrá et al., 2014).

Based on the results by several studies on llama dry-cured sausages and as expected, the environmental and production conditions have a remarkable influence on the product bacterial composition, e.g., the occurrence of predominant species selected during the process was found in pilot but not in many artisanal productions, in which the same bacteria present at start where equally present at the end (Cocconcelli et al., 2012). Several genera and species were found in diverse studies, which are listed in Table 1. As indicated in them, *Lactobacillus sakei* seems to be a robust LAB species that is very well adapted to the harsh matrix and process conditions, strains of which have the potential to be developed as starter cultures for fermented meat products.

#### *Charqui* (*charque*)

*Charqui* or *charque*, from the Quechua language “ch’arki,” is a type of salted and sun-dried meat of intermediate moisture with a shelf-life of months at room temperature. The broader category of this fermented meat incorporates *charqui* meat itself and its derivatives, jerked beef and *carne-de-sol* (sun meat; Shimokomaki et al., 2016). *Charqui* meats are typical of the Andean region, of Argentina, Chile, Peru, Bolivia and also Brazil. It has been consumed since pre-Columbian times to preserve meat for long periods. Historically, guanaco or llama meat was used (Figure 2), but with the arrival of the Spaniards in America, *charqui* made from beef became more common (Fadda and Vignolo, 2014).

For modern production, deboned raw meat muscles are injected with a brine solution, coated with coarse marine salt and piled up to let juices drain out. The piles are inverted every 24 h for several days, and in the final step, the salted meat pieces are exposed to the sun on rails until water activity (a_w_) decreases to 0.7–0.8. The process can take up to 10 days. Due to the low a_w_, the proliferation of pathogenic bacteria is unlikely, but halophilic and halotolerant bacteria may be able to grow. These include both bacteria favouring the fermentative process as well as spoilage bacteria, such as *Halobacterium cutirubrum*, which causes changes in odour and the appearance of slime and red spots on the product surface, coming from the salt used in the process (Pinto et al., 2002; Youssef et al., 2007). The fermentative process carried out by autochthonous microorganisms, mainly *Micrococcus* spp., *Pediococcus halophilus, Staphylococcus xylosus,* Lactobacilli*. and Streptococcus* spp. is essential for the unique sensorial characteristics of *charqui,* proteolytic activity and production of bacteriocins (Pinto et al., 2002).

### Starchy products

Andean roots and tubers constitute traditional energy sources due to their high content of starch, but also can be rich in fructooligosaccharides (FOS), glucosinolates, antioxidants or other bioactive ingredients (Leidi et al., 2018). The granular nature of starch makes it hard to metabolize because the enzymes cannot access the interior of the granules. However, some microorganisms can metabolise starch granules in more or less complex sugars due to the availability of different amylolytic enzymes.

South American crops include a diverse variety of potatoes (*Solanum tuberosum*), oca (*Oxalis tuberosa*), papalisa (*Ullucus tuberosus*), yacon (*Smallanthus sonchifolius*), ahipa (*Pachyrhizus ahipa*), arracacha (*Arracacia xanthorrhiza*), mashua (*Tropaeolum tuberosum*), yam (*Discorea* sp.), cassava (*Manihot esculenta* Crantz), also called yuca or manioc and maize (*Zea mays*). These crops have been used as substrates for the production of fermented products such as *tocosh*, *chicha*, *tiquira* (cassava spirit), *polvilho azedo* (sour powder), *masa agria* and others through traditional techniques (Ray and Didier, 2015; Mosso et al., 2016; Jiménez et al., 2018; Leidi et al., 2018).

#### Tocosh

*Tocosh*, togosh, or *fute* is a traditional fermented food product that is still prepared in small communities in the highlands of Central Peruvian Andes and the south of Colombia. It is usually prepared with potatoes, but other plants such as maize, papalisa, oca, or arracacha are also used. However, in this review we will refer to the preparation of potato-based *tocosh*. The traditional process consists of digging a ground hole near a water spring in which large amounts of potatoes (normally discarded potatoes) are placed between straw layers (*shicshi*). Then, the pile is covered with rocks in order to prevent the tubers from being washed away by the slight water current; the potatoes are left to ferment in this running water for 1–12 months. During this time, potatoes undergo an enzymatic blackening or browning and are laid in a dry shaded area to drain the water. Once dried, the product can be stored for consumption or sale. However, most commonly, the product is sun-dried and ground to obtain a fine flour-type product that is used to prepare broths, stews and *mazamorra de tocosh*, which is prepared by boiling a mixture of *tocosh*, sugar, cinnamon, cloves and water, and then served cold (Ray and Didier, 2015; Jiménez et al., 2018).

Jiménez et al. (2018) investigated the LAB diversity associated with potato *tocosh* for the first time, using culturing and HTS approaches. The study was performed on freshly harvested potatoes, as well as 1- and 8-month fermented samples. *Lactobacillus* species (*L. sakei, L. casei, Lactobacillus farciminis, Levilactobacillus. brevis, L. fermentum*) and *Leu. mesenteroides* predominated on fresh potatoes, however *Clostridium*, *Zymophilus* and *Prevotella* genus were predominantly present in 1- and 8-months *tocosh* samples. Some LAB were examined in greater depth, with several strains of *L. sakei* and *Leu. mesenteroides* displaying amylase and phytate-degrading abilities, as well as EPS, group B vitamin (riboflavin and folate) production capacity and antibacterial activity (Jiménez et al., 2018). Yépez et al. (2017) also characterised the LAB strains from *tocosh* from the perspective of their antibacterial and antifungal activity against spoilage microbes, and the responsible antimicrobial compounds from the supernatant. They found that *L. fermentum* T3M3 and *Leu. mesenteroides* T1M3 have potential as antimicrobial producers by inhibiting bacterial foodborne pathogens and spoiler fungi directly as well as the activity of their cell-free supernatant activity against toxigenic fungi (Yépez et al., 2017). Yeasts and moulds were also counted, decreasing from 7.1 × 10^3^ (fresh potatoes) to 1.0 × 10^2^ CFU/g (8-months sample).

Finally, yet another study, focused on *tocosh* from two varieties of potato, *Yungay* and *Peruanita*, the authors reported that viable mesophilic aerobic microorganisms increased from 1.8 × 10^4^ to 3.6 × 10^7^ cfu g^−1^, fungi and yeasts from 2.4 × 10^2^ to 4.4 × 10^3^ cfu g^−1^ decreasing in the final stage to 5.0 × 10^1^ cfu g^−1^. LAB counts ranged from 1.5 × 10^4^ to 6.6 × 10^8^, with *L. brevis*-*Lactobacillus acidophilus* increasing from 1 × 10^2^ to 3.6 × 10^5^ cfu g^−1^ (Yábar and Reyes, 2017).

These few studies suggest that *Lactobacillus* and *Levilactobacillus* genera play an important role in *tocosh* fermentation. It is also probable that the organic acids and antimicrobials produced during the process are the responsible for the decrease in the fungal population, including toxigenic fungi, leading to a stabilization of the final product, as observed on many fermented foods.

#### Masa agria

*Masa agria* is a maize fermented dough made in a traditional way in Colombia. To prepare *masa agria*, the kernels of yellow maize are covered with water and buried in a hot place (35°C–40°C) for 4 days, before the kernels are washed, milled and dehydrated for 3 or 5 days. The quality and sensory characteristics depend of the fermentation conditions such as the fermentation time and the different microorganisms present (Chaves-López et al., 2014b). The *masa agria* produced is used to prepare different traditional products such as tamales, empanadas, carantanta among others (Chaves-Lopez et al., 2016).

Chaves-Lopez et al. (2016) studied six samples of commercial *masa agria* from different producers from Valle del Cauca (Colombia). They used HTS to study the microbial composition and found that the genus *Lactobacillus* (mainly *L. fermentum*) was most abundant, followed by *Acetobacter* and *Weissella*. The abundance of these microorganisms is also responsible for the low pH (3.1–3.7) registered. *L. fermentum* seems to be a key organism for fermentation of maize, making the large amounts of starch available to the overall community. The fermentation products (lactate, formate and ethanol) may also serve as carbon sources for organisms, such as yeasts.

#### Fermented products made from cassava roots

Cassava derivatives form part of Brazilian ethnic foods and culture, such as, *puba*, *tiquira* spirit, *carimã*, *farinha d’água* and *polvilho azedo*. Moulds and bacteria (mainly LAB) are the first to colonise raw cassava, metabolising the pre-existing sugar. The traditional methods of preparing food and beverages made from cassava rely on enzymes produced by moulds and naturally present bacteria, which hydrolyse the starch, producing fermentable sugars, as well as the yeasts that ferment these sugars to ethanol.

##### Beiju

*Beiju*, a traditional product from the Brazilian state of Maranhão is an example of a fermented cassava product. After being peeled and grated, the cassava is pressed, and the liquid is discarded. The resulting dough is used to make large *beijus*, which are toasted in an oven plate, periodically sprinkled with a little water on both sides, stretched over and coated with banana or palm leaves. After 3–4 days, *beijus* covered in fungi are placed in large closed containers. After 2 more days, *beijus* are moist, with a clear, yellowish liquid that runs out of them. At this point, *beijus* are ready for use, but they can also be dried and stored for long periods (Chuzel and Cereda, 1995; Penna et al., 2017).

Across different studies on *beijus* samples, a variety of fungal strains of the genera *Paecilomyces*, Aspergillus, *Neurospora*, among others, were isolated and studied, showing high α-amylase and amyloglucosidase activities. Additionally, other studies suggest *Neurospora crassa* and the pink-coloured *Manila sitophila* showed strong amylolytic activity indicating these moulds are also agents responsible for starch saccharification (Park et al., 1982; Chuzel and Cereda, 1995; Penna et al., 2017). Regarding the formation of aromas, *Neurospora* isolates were found to have a pleasant fruity aroma that is attributed to ethyl hexanoate (Park et al., 1982). More studies on the microbiota of cassava and *beijus* need to be carried out to better understand this process, since other non-yet characterised microorganisms could be involved. However, the few studies carried until now, indicate that different mould species are highly active and key microorganisms in *beiju* fermentation, which agrees with the extraordinary starch-degrading and transforming enzymes that fungi possess and are necessary for carbohydrate availability and further fermentation.

##### Puba flour

The manufacturing of *puba* is based on ancient empirical knowledge of indigenous Brazilian tribes, whose traditional methods of preparation have hardly changed over time. Currently, *puba flour* serves as a staple food for much of the Brazilian population, particularly for inhabitants of the north and northeast regions. To prepare *puba*, unpeeled manioc roots are directly steeped in stagnant water or are placed in a bag kept in running water at room temperature for a period of 3–7 days. Otherwise, the roots may be left in the sun or in ovens to produce dry *puba* flour, which has a moisture content of ~13%. Either the wet or dry *puba* may be used to prepare couscous, cassava paste porridge and a variety of *puba* cakes. Although there is little available data on the microbiota involved in the fermentation of *puba* in Brazil, it has been well documented that microorganisms associated with submerged fermentation of cassava include LAB, *Enterobacteriaceae*, *Corynebacterium* sp. *Bacillus* sp., *Clostridium* sp., yeasts and moulds. LAB predominate during the whole process and their metabolic activity contributes to the production of organic acids, the extension of the shelf life and safety of the final product by delaying microbial spoilage or inhibiting microbial pathogens and the development of desirable sensory characteristics (flavour, visual appearance, texture; Padonou et al., 2010; Anyogu et al., 2014; Fayemi and Ojokoh, 2014; Penna et al., 2017). Some studies indicate that LAB present in *puba* flour are usually found in two to four species associations, which suggests a role for synergistic phenomena, similar to that found in studies on African fermented cassava products *lafun* and *foo-foo* using molecular methods, in which *L. fermentum* was also a key component (Lacerda et al., 2005; Crispim et al., 2013; Penna et al., 2017).

## Conclusion

This review highlights the diverse microbial composition amongst fermented foods of South American origin that have been investigated to date. However, despite the great variety of fermented foods available in South America several research gaps remain in fully understanding these foods from a microbial perspective. There remain many traditional fermented foods from South America that have yet to be investigated to determine their microbial composition. Where research has been completed, the majority of these foods have only been investigated in 1 or 2 studies and so potentially not reflective of all samples of these food types. Additionally, only enumeration information is available in the majority of cases with the absence of microbial activity within the fermentation processes. Understanding of these processes would be incredibly useful in future investigations and could offer a more thorough comprehension of these fermented foods. Through the development of an even greater understanding of the microbial populations present and their contributions to the characteristics of these foods, it will be possible to harness this knowledge to improve these fermented foods to optimise quality, safety and health-promoting attributes.

In the research published to date, as with many fermented foods, LAB and yeasts are important components of fermented foods of South American origin, but bacteria belonging to the *Bacillus* genus also have an essential role in the production of fermented beverages. Although the role of individual microbial members has not yet been determined for those fermented foods discussed, from other fermented foods we know LAB have proteolytic activity important for flavour production within the final product while also producing other metabolites, *Bacillus* have amylases, lipases, cellulases and proteases activities, while yeast are primarily involved with CO_2_ and ethanol production with some other metabolites also produced (Bintsis, 2018; Rakhmanova, et al., 2018; Maicas, 2020).

Fermented beverages and dairy foods share the greatest number of microorganisms, potentially as many of the fermented beverages are also of dairy origin. Investigation of fermented foods more broadly has identified a variation in species observed on the basis of substrate previously (Leech et al., 2020). *Lactobacillus* species in general and the species *L. plantarum* in particular were identified across all fermented food types, highlighting their importance and relevance in fermented food systems. Overall, there are many traditional and artisanal fermented foods produced throughout South America from a variety of substrates. The microbial composition of these products has not been yet fully elucidated, but some efforts have been made in understanding the microbial contributions to these fermentation processes. As many of these fermented foods are unique to South America it would be of great interest to fully understand how they vary from fermented foods of other origins with similar substrates.

## Author contributions

MJ, CO’D, and MU designed and wrote the manuscript. PC critically revised the manuscript. All authors contributed to the article and approved the submitted version.

## Funding

This project has been supported by the European Union’s Horizon 2020 research and innovation programme under the Marie Sklodowska-Curie grant agreement no. 754535.

## Conflict of interest

The authors declare that the research was conducted in the absence of any commercial or financial relationships that could be construed as a potential conflict of interest.

The reviewer RH declared a past co-authorship with one of the author PC to the handling editor.

## Publisher’s note

All claims expressed in this article are solely those of the authors and do not necessarily represent those of their affiliated organizations, or those of the publisher, the editors and the reviewers. Any product that may be evaluated in this article, or claim that may be made by its manufacturer, is not guaranteed or endorsed by the publisher.
